# 4-Octyl itaconate attenuates LPS-induced acute kidney injury by activating Nrf2 and inhibiting STAT3 signaling

**DOI:** 10.1186/s10020-023-00631-8

**Published:** 2023-04-24

**Authors:** Lujun Xu, Juan Cai, Chenrui Li, Ming Yang, Tongyue Duan, Qing Zhao, Yiyun Xi, Liya Sun, Liyu He, Chengyuan Tang, Lin Sun

**Affiliations:** Department of Nephrology, Key Laboratory of Kidney Disease and Blood Purification, The Second Xiangya Hospital of Central South University, No.139 Renmin Middle Road, Changsha, Hunan 410011 China

**Keywords:** sepsis, Acute kidney injury, 4-octyl itaconate, Oxidative stress, Inflammation, Mitophagy, Nrf2, STAT3

## Abstract

**Background:**

Septic acute kidney injury (S-AKI) is the leading form of acute kidney failure among hospitalized patients, and the inflammatory response is involved in this process. 4-octyl itaconate (4-OI) is a multi-target itaconate derivative with potent anti-inflammatory action. However, it remains elusive whether and how 4-OI contributes to the regulation of S-AKI.

**Methods:**

We employed a lipopolysaccharide (LPS)-induced AKI murine model and explored the potential renoprotective effect of 4-OI *in vivo*. *In vitro* experiments, BUMPT cells, a murine renal tubular cell line, were conducted to examine the effects of 4-OI on inflammation, oxidative stress, and mitophagy. Moreover, STAT3 plasmid was transfected in BUMPT cells to investigate the role of STAT3 signaling in the 4-OI-administrated state.

**Results:**

We demonstrate that 4-OI protects against S-AKI through suppressing inflammation and oxidative stress and enhancing mitophagy. 4-OI significantly reduced the levels of Scr, BUN, Ngal as well as the tubular injury in LPS-induced AKI mice. 4-OI restrained inflammation by reducing macrophage infiltration and suppressing the expression of IL-1β and NLRP3 in the septic kidney. 4-OI also reduced ROS levels, as well as cleaved caspase-3 and boosted antioxidants such as HO-1, and NQO1 in mice. In addition, the 4-OI treatment significantly promoted mitophagy. Mechanistically, 4-OI activated Nrf2 signaling and suppressed phosphorylated STAT3 in vivo and vitro. Molecular docking revealed the binding affinity of 4-OI towards STAT3. ML385, a specific Nrf2 inhibitor, partially repressed the anti-inflammatory and anti-oxidative effects of 4-OI and partially restricted the mitophagy induced by 4-OI *in vivo* and *in vitro*. Transfected with STAT3 plasmid partially suppressed mitophagy and the anti-inflammatory effect provoked by 4-OI *in vitro*.

**Conclusion:**

These data suggest that 4-OI ameliorates LPS-induced AKI by suppressing inflammation and oxidative stress and enhancing mitophagy through the overactivation of the Nrf2 signaling pathway, and inactivation of STAT3. Our study identifies 4-OI as a promising pharmacologic for S-AKI.

**Supplementary Information:**

The online version contains supplementary material available at 10.1186/s10020-023-00631-8.

## Introduction

Acute kidney injury (AKI), a condition with rapidly reduced renal function, characterized by a reduction of GFR or oliguria, remains a major contributor to kidney dysfunction. Septic acute kidney injury (S-AKI) is the leading form of acute kidney failure. And the global incidence of S-AKI might be approximately 6 million cases per year [1]. S-AKI is found in about 40–50% of patients in the ICU [2, 3], which associates with high mortality and leads to adverse long-term outcomes [4]. Unfortunately, there are still no effective strategies for the treatment of S-AKI.

Recent studies show that lipopolysaccharide (LPS) mediated inflammation and oxidative stress play a partial role in the kidney injury of S-AKI [5, 6]. LPS is in the outer membrane of gram-negative bacteria [6], mediating inflammation through triggering the TLR4 complex of immune cells [7], endothelial cells [8], and tubular cells [9] of the kidneys, releasing a series of cytokines, such as interleukin (IL)-1, tumor necrosis factor (TNF)-α and IL-6. LPS-induced AKI is the most common model to analyze S-AKI [10]. Moreover, mitochondria dysfunction leads to the imbalance between reactive oxygen species (ROS) and the reduction system, resulting in oxidative stress in response to inflammation in sepsis [11–14]. Emerging data have indicated that repression of inflammation and oxidative stress has a positive effect on the treatment of S-AKI [15], but the precise mechanism is largely unknown. Additionally, in LPS-induced S-AKI, Tran et al. observed mitochondrial swelling and disruption of cristae in renal tubular cells, which are associated with mitochondrial dysfunction [16]. Mitophagy is a process of selectively degrading damaged mitochondria under mitochondrial toxicity conditions, which plays an essential role in mitochondrial quality control. Recent studies show that mitophagy plays a pivotal role in the kidney injury of S-AKI [9, 17]. In a rat model of S-AKI, mitophagy in proximal renal tubules was induced at 12 h after induction, and bone marrow-derived mesenchymal stem cells (BMSCs) were shown to ameliorate tubular cell injury by promoting mitophagy [18]. Furthermore, wang and colleagues showed that the PINK1/PARK2 pathway-mediated-mitophagy plays an important role in mitochondrial quality control in the S-AKI mice [9].

4-octyl itaconate (4-OI) is a derivant of itaconate with better cell-permeable and can be degraded into itaconate in vivo [19]. Recently, many studies suggested that 4-OI plays an immunomodulatory role in a range of diseases via upregulating Nrf2 signaling in macrophages [19, 20], or hepatocytes [21]. Moreover, a link or association between 4-OI and mitochondrial function has been indicated. For example, 4-OI can be degraded into itaconate in vivo, and itaconate is an inhibitor of succinate dehydrogenase (SDH) of the mitochondrion in microglia [22] and macrophages [23]. However, the effect of 4-OI and the underlying mechanism in S-AKI remain largely unclear.

Herein, we demonstrate that 4-OI protects against oxidative stress and inflammation via Nrf2 signaling in LPS-induced acute kidney injury. Moreover, our data provide new insights into the mechanisms triggering mitophagy by 4-OI modulated by Nrf2 and STAT3 in vitro and vivo, and STAT3 may be a potential target of 4-OI.

## Materials and methods

### Antibodies and reagents

The antibodies used in this study were as follows: anti-F4/80 (GB11027), anti -SQSTM1 / p62 (GB11531) from Servicebio; anti-cleaved caspase-3 (9661 S), anti- Phospho-Stat3 (Tyr705) (9145 S) from Cell Signaling Technology; anti-IL-1β (16806-1-AP), anti-Nrf2 (16396-1-AP), anti-β-actin (20536-1-AP), anti-HO-1 (27282-1-AP), anti-BAX (60267-1-Ig), anti-NQO1 (67240-1-Ig), anti-STAT3 (60199-1-Ig), anti-TIMM23 (67535-1-Ig), anti-TOMM20 (66777-1-Ig) from Proteintech; anti-NLRP3 (ab263899), anti-LC3B (ab192890), anti-STAT3 (phospho S727) (ab32143) from Abcam, anti-LC3β (sc-271,625) from Santa Cruz Biotechnology. LPS (Escherichia coli, serotype 0111: B4; L413000) and (2-hydroxypropyl)-β-cyclodextrin (H107) were purchased from Sigma-Aldrich. 4-OI was purchased from Cayman Chemical (Item No. 25,374).ML385 was purchased from Selleckchem (Catalog No. S8790). MitoQ was obtained from FOCUS Biomolecules.

### Mouse model

Male C57BL/6 mice (8-week-old) were purchased from Hunan Slack King Experimental Animal Company. Animals were fed a normal chow diet with free access to water and housed in a room with a 12-hour light/dark cycle and an ambient temperature of 22 °C. Animals were randomized into five groups, as below: control group, 4-OI group, LPS group, LPS + 4-OI group, and LPS + 4-OI + ML385 group. For LPS-induced AKI models, LPS at a dose of 10 mg/kg was intraperitoneally (i.p.) injected into the mice [10]. AKI is defined as a 50% increase in baseline serum creatinine (Scr) within 48 h according to the Kidney Disease Improving Global Outcomes (KDIGO) [24]. 4-OI (50 mg/kg/dose, i.p.) was given 2 h before LPS treatment [19]. ML385 (dissolved in DMSO,30 mg/kg/dose, i.p.) was given 2 h before LPS administration [25]. 4-OI should be dissolved in DMSO and then in (2-hydroxypropyl)-β-cyclodextrin [21]. The control mice were injected with the same amount of 0.9% saline and the vehicle solution. All animals were anesthetized with pentobarbital and sacrificed at 24 h after LPS injection. Blood and kidney tissues were harvested for further experiments. All the experiments in this study were approved by the Medical Ethics Committee of Central South University and followed the NIH guidelines for the care and use of laboratory animals.

### Cell culture and chemical treatment

The Boston University mouse proximal tubular epithelial cell line (BUMPT) was gifted by Professor Dong Zheng from the Nephrology Institute of the Second Xiangya Hospital of Central South University. The cells were cultured in the DMEM medium containing 10% fetal bovine serum (FBS). For LPS treatment, BUMPT cells at a confluence of 50–60% were incubated in a DMEM medium with 0.2% FBS for 24 h. Then, the cells were treated with DMEM medium containing 0.2% FBS plus 100 ug/ml LPS for 24 h. To examine the effect of 4-OI, 4-OI was added 2 h before LPS treatment. To determine the effect of the Nrf2 inhibitor, ML385 (10 umol/l) was added together with 4-OI. To determine the effect of MitoQ, MitoQ (100 nM) was added together with 4-OI and ML385. Furthermore, cells were transfected with a pLV3-STAT3-flag plasmid (synthesized from MiaoLing Plasmid Platform) using a Lipofectamine 3000 reagent (Invitrogen) according to the manufacturer’s instructions.

### Assessment of metabolic parameters

Scr was determined using a Creatinine Assay kit (sarcosine oxidase) (C011-2-1, Nanjing Jiancheng Bioengineering Institute) following the manufacturer’s instructions. Blood urea nitrogen (BUN) was measured with reagents from BioAssay Systems (DIUR-100) according to the manufacturer’s directions.

### Renal histology

Renal tissue samples were harvested, sliced into 4 um thick sections, and processed for hematoxylin and eosin (H&E) staining as previously described [10]. Damaged renal tubules were characterized by the following changes: brush border loss, tubular dilation and disruption, formation of the cast, and cell lysis. Tissue damage was scored in a blinded manner by the percentage of damaged renal tubules [9].

### Transmission electron microscopy (TEM)

The kidney cortex tissues were fixed with 2.5% glutaraldehyde and then treated with standard procedures, including dehydration, osmosis, embedding, sectioning, and staining. The ultrastructure of renal cells was observed using a Hitachi H7700 electron microscope.

### Real-time q-PCR

The total RNA of BUMPT cells was extracted using RNAex Pro Reagent (Accurate Biology, AG21101). Then, RNA was reverse-transcribed to cDNA using Evo M-MLV One-Step RT-qPCR Kit (Accurate Biology, AG11708). Subsequently, qPCR was performed using a 2 x SYBR® Green Pro Taq HS Premix (Accurate Biology, AG11701) with a 7300 Real-Time PCR System (Applied Biosystems) as previously described [26]. Primers are listed in Table 1 [27].

### Western blot analysis

BUMPT cells and renal cortical tissues were lysed using RIPA buffer (CWBIO) containing protease inhibitors and phosphatase inhibitors (CWBIO). Protein concentration was determined with the BCA assay kit (Thermo Fisher Scientific). Denatured proteins were analyzed by sodium dodecyl sulfate-polyacrylamide gel electrophoresis (SDS-PAGE) and then transferred onto a polyvinylidene difluoride membrane at 290 mA for 110 min. Subsequently, blots were blocked with 5% fat-free milk for 2 h and probed subsequently with primary antibodies overnight. Membranes were washed three times in tris (hydroxymethyl) aminomethane-buffered saline with Tween 20 (TBST) for 10 min and immersed in solutions containing secondary antibodies for 1 h. The bands were washed in TBST for 30 min before being evaluated by a Tanon 5200 Multi-instrument (Tanon Instruments, China). The density of the band was analyzed using Image-J software.

### Analysis of ROS production

A dihydroethidium (DHE) (Invitrogen) probe was used to evaluate intracellular ROS production in renal tissues. MitoSox Red (Invitrogen) was used to evaluate the generation of mitochondrial ROS of BUMPT cells.

### Immunohistochemistry (IHC) staining

Paraffin-embedded kidney sections were deparaffinized and rehydrated using slide warmers and alcohol. And then the antigen was retrieved with citrate buffer for 60 min. All sections were incubated in 0.3% hydrogen peroxide for 10 min to block the endogenous peroxidase. The sections were blocked with 5% bovine serum albumin (BSA). The slides were then exposed to primary antibodies at 4 ℃ overnight. After washing, the sections were incubated with a secondary antibody for 20 min at room temperature, reacted with diaminobenzidine using a DAB kit (Servicebio), and stained with hematoxylin. The sections were examined by light microscopy.

### Immunofluorescence (IF) staining

The above-described kidney sections were used for IF staining according to previously published procedures [28]. Briefly, the sections were labeled with the primary antibody and the corresponding secondary antibody. For cell immunofluorescence, cells were washed with phosphate-buffered saline (PBS) and fixed in pre-cold methanol for 10 min at – 20 ℃. After washing with PBS, the fixed cells were blocked in 10% normal goat serum for 1 h. Cells were incubated with antibodies overnight at 4 ℃, followed by incubation with secondary antibodies for 1 h at 37 ℃. Then, the cells were stained with DAPI to indicate nuclei. Finally, the cells were examined with a microscope (Olympus Corporation, Tokyo, Japan).

### Analysis of apoptosis

For Hochest 33258 staining, progress was achieved by following the manufacturer’s instructions. Briefly, the cells that were grown on Bioflex plates were incubated with fixation buffer (0.5 ml per well) for 10 min at room temperature and washed twice with PBS. Then, the cells were stained with 1 ug/mL Hochest 33258(Invitrogen, H3569) for 10 min in the dark, washed twice with PBS, and examined by fluorescence microscopy. The Hoechst reagent was taken up by the nuclei of the cells, and apoptotic cells exhibited bright blue fluorescent, condensed, and fragmented nuclei. The apoptotic index (AI) was calculated as the percentage of apoptotic nuclei per total nuclei number per field as ref [29].

### 13. Molecular docking

The crystal structure of STAT3 was obtained from the PDB database (http://www.pdb.org) and was then imported into Pymol software (version 2.4.0) for the removal of water molecules, co-crystallized ligands, and ions. Subsequently, hydrogens were added and gasteiger charges were computed by AutoDock Tools (version 1.5.6). The structures were saved as PDBQT format files after the AD4 type was assigned. 3D-structure of 4-OI was downloaded from PubChem database (https://pubchem.ncbi.nlm.nih.gov/) and was converted into Mol2 format files by Open Babel GUI software (version 2.3.1) http://openbabel.org) [30]. Then the small molecule ligand file was imported into AutoDock Tools (version 1.5.6) and torsions were set automatically. The structures were then saved as PDBQT format files, too. Subsequently, PDBQT files of macromolecule receptors and corresponding small molecule ligands were imported into AutoDock Tools (version 1.5.6) for the construction of mating pockets. Further molecular docking simulation was conducted in AutoDock Tools (version 1.5.6) by using a Genetic algorithm. Autodock Vina (version 1.1.2) was used for docking the receptor protein with the small molecule ligands binding energy (DG in kcal/mol). Analysis and visualization were performed using PyMOL (version 2.4.0).

### Statistics

Statistical differences in multiple groups were determined by multiple comparisons with ANOVA followed by a post-hoc test [31]. The Kolmogorov-Smirnov test was used to determine whether the data were consistent with a Gaussian distribution. Data were presented as the means ± SD. SPSS 22.0 and GraphPad Prism 7.0 were used for statistical analysis. All tests were two-tailed and a p-value ≤ 0.05 was considered statistically significant.

## Results

### 4-OI ameliorates LPS-induced nephrotoxicity *in vivo* and apoptosis of tubular cells *in vitro*

**Fig. 1:**
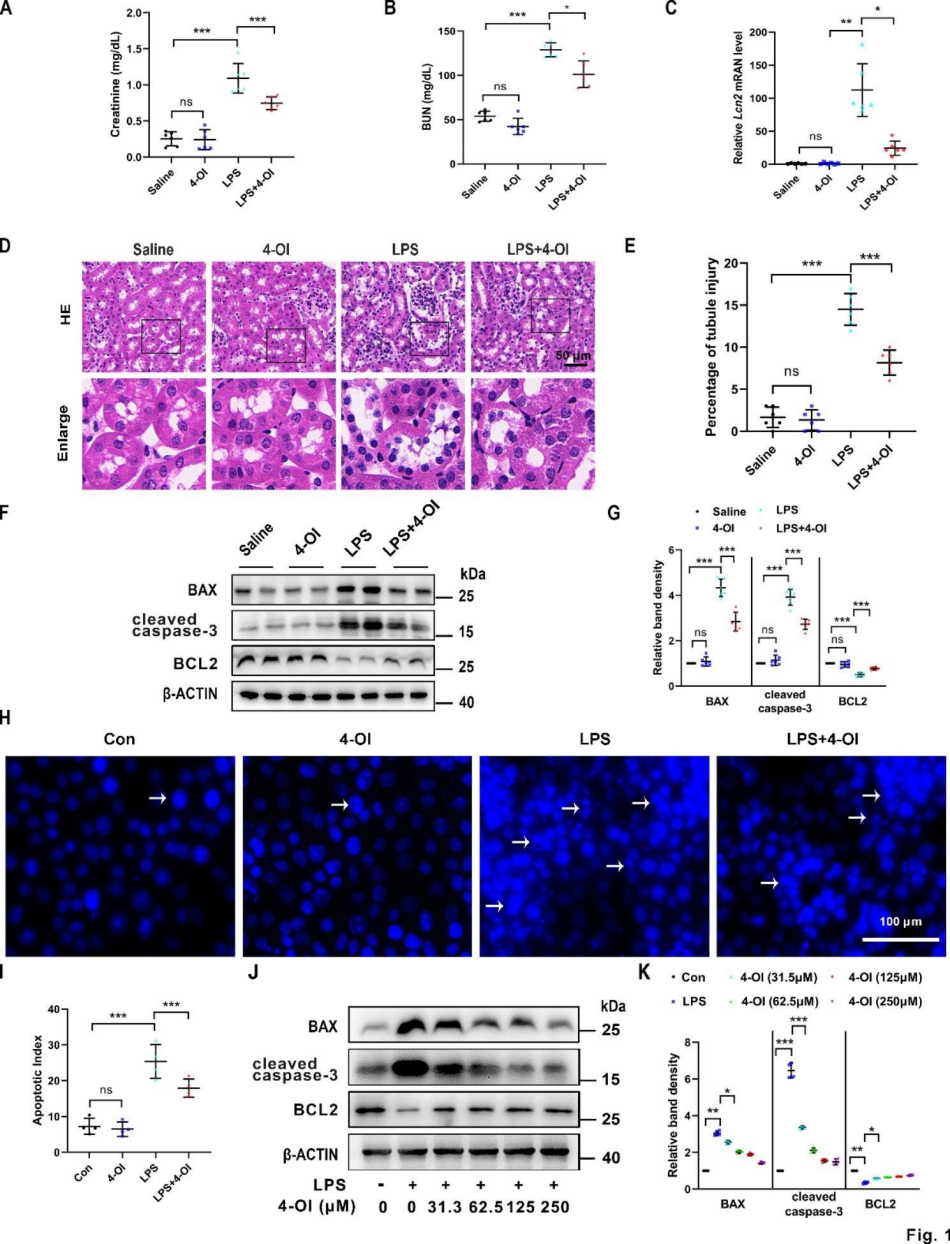
**4-OI decreases LPS-induced nephrotoxicity in vivo and apoptosis in vitro**. Scr (**A**) and BUN (**B**) levels of different groups of mice exposed to LPS or 4-OI (n = 6). (**C**) qPCR analysis of mRNA of *Lcn2 * in renal tissues (n = 6). (**D**) Representative images of hematoxylin and eosin (H&E) staining of the renal cortex. Vacuolization and loss of brush border in tubular cells are pointed to by the arrows. Scale bar: 50 μm. (**E**) Tubular injury is evaluated by figuring out the renal tubules with signs of injury (n = 6). (**F**) Representative western blot bands of BAX, cleaved caspase-3, BCL2, and β-actin (protein loading control) in renal cortex tissues. **(G)** Relative band density of BAX, cleaved caspase-3 and BCL2 signals (n = 6). **(H)** In Hoechst 33258 staining images, nuclear condensation, fragmentation, and apoptotic bodies were observed. Scale bar = 100 μm. **(I)** The apoptotic index (AI) was calculated as the percentage of apoptotic nuclei per total nuclei number per field. Representative western blot bands **(J)** and relative band intensity **(K)** of BAX, cleaved caspase-3 and BCL2 in BUMPT cells treated with different doses of 4-OI under saline or LPS conditions (n = 4). Each symbol (circle) represents an independent experiment. Data are presented as means ± SDs. *P < 0.05, **P < 0.01, ***P < 0.001; ns, not significant. LPS, lipopolysaccharide; 4-OI, 4-octyl itaconate; Scr, serum creatinine; BUN, blood urea nitrogen; qPCR, Real-time polymerase chain reaction

We first assessed the biochemical indexes and renal morphological alterations in LPS (the main endotoxin in sepsis)-induced AKI mice treated with or without 4-OI. Compared to LPS treated only, additional 4-OI significantly reduced levels of serum creatinine (Scr) (Fig. 1A) and blood urea nitrogen (BUN) (Fig. 1B) in the murine model. In addition, quantitative polymerase chain reaction (qPCR) revealed that 4-OI treatment partially diminished the increased *Lcn2*(Ngal) mRNA level induced by LPS (Fig. 1C). The tubular damages, characterized by the vacuolization and loss of brush border in tubular cells, formation of tubular casts, sloughing of cells into the tubular lumen, and leukocyte infiltration, were markedly reduced in the renal cortex of the LPS + 4-OI group compared to the LPS group (Fig. 1D). The results were further corroborated by quantitative analysis of the tubular damage in the kidneys (Fig. 1E). Meanwhile, immunoblotting revealed that the apoptotic markers, BAX (Fig. 1F and G) and cleaved caspase-3 (Fig. 1F and G) induced by LPS, were suppressed in the renal cortex of AKI mice administrated with 4-OI. Simultaneously, the protein level of BCL2 was increased in the LPS + 4-OI group compared to the LPS-treated group (Fig. 1F and G). Moreover, BUMPT cells incubated with LPS for 24 h, exhibited obvious morphological changes characterized by nuclear condensation, fragmentation, and apoptotic bodies, which suggested the occurrence of apoptosis. And the percentage of apoptotic cells was restored by 4-OI treatment in the BUMPT cells (Fig. 1H and I). Immunoblot of cleaved caspase-3, BAX, and BCL2 also showed 4-OI suppressed the cell death in a dose-dependent manner in BUMPT cells (31.5 to 250 μm) (Fig. 1J K). Collectively, these data indicate that 4-OI protected against LPS-induced nephrotoxicity* in vivo* and apoptosis of tubular cells* in vitro*.

### 4-OI alleviates LPS-induced renal oxidative stress and inflammation in mice and BUMPT cells

**Fig. 2:**
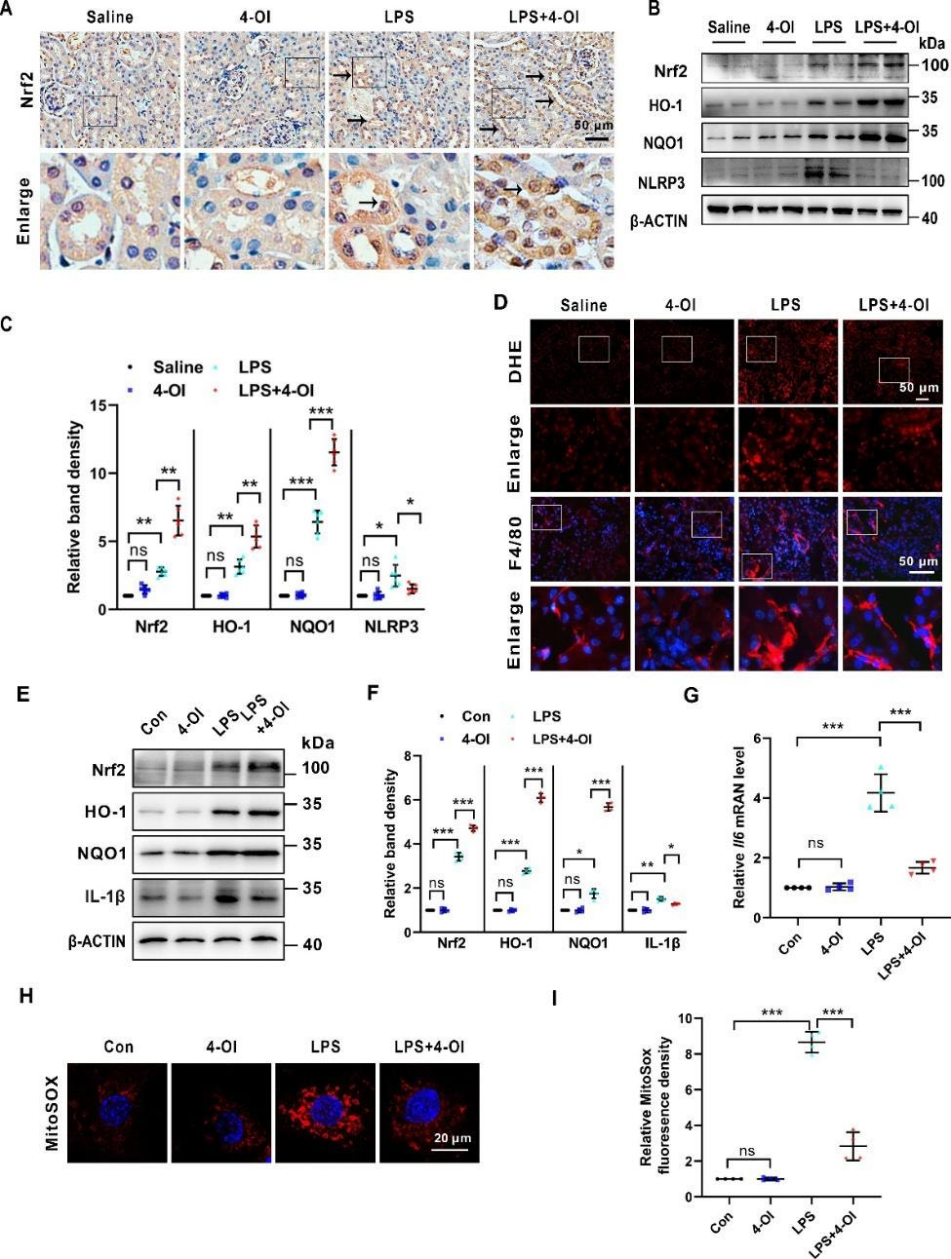
**4-OI activates Nrf2 and alleviates inflammation and oxidative stress in LPS-induced AKI *in vivo *and *in vitro***. **(A)** Representative IHC staining images of Nrf2 (pointed to by the arrowhead) of renal tissues. Scale bar: 50 μm. **(B)** Representative western blot bands of Nrf2, HO-1, NQO1, and NLRP3 in renal cortex. **(C)** Relative band density of Nrf2, HO-1, NQO1 and NLRP3 in kidney cortex (n = 6). **(D)** Representative DHE staining images and IF staining images of F4/80. Scale bar: 50 μm. **(E)** Representative western blot bands of Nrf2, HO-1, NQO1, and IL-1β in BUMPT cells. **(F)** Relative band density of Nrf2, HO-1, NQO1, and IL-1β in BUMPT cells (n = 4). **(G) **qPCR analysis of mRNA of *Il6* in BUMPT cells (n = 4). **(H) **Representative images of MitoSOX staining in BUMPT cells. Scale bar: 20 μm. **(I)** Semi-quantification analysis of MitoSOX fluorescence intensity. Each symbol (circle) represents an independent experiment. Data are presented as means ± SDs. *P < 0.05, **P < 0.01, ***P < 0.001; ns, not significant. IHC, Immunohistochemistry; IF, immunofluorescence; DHE, dihydroethidium; Nrf2, nuclear factor (erythroid-derived 2)-like 2; HO-1, heme oxygenase-1; NQO1, NAD(P)H quinone oxidoreductase 1

As inflammation and oxidative stress play a critical role in the progression of S-AKI, and Nrf2 signaling is a key regulator to these processes [32], we examined their alterations in the renal tissues and BUMPT cells subjected to LPS with or without 4-OI. As shown in Fig. 2A, compared to the LPS group, the administration of 4-OI to S-AKI mice notably increased the expression and nuclear translocation of Nrf2 in the renal cortex by immunohistochemistry (IHC) staining. Western blot analysis further confirmed the elevated Nrf2 protein levels of the renal cortex of mice pre-treated with 4-OI compared to the LPS group (Fig. 2B C). In addition, immunoblot analysis demonstrated that 4-OI enhanced the increased expression of HO-1, and NQO1, two anti-oxidative enzymes, in the LPS-treated renal cortex of mice. In parallel, the oxidative stress assessed by dihydroethidium (DHE) staining induced by LPS was reduced by 4-OI treatment (Fig. 2D). Furthermore, the western blot demonstrated that 4-OI reversed the increased level of NLRP3, an indicator of inflammation, induced by LPS in murine kidneys (Fig. 2B C). Immunofluorescence (IF) staining of F4/80 indicated infiltration of macrophages in LPS-treated kidneys, and the number of infiltrated macrophages was significantly decreased by 4-OI administration in LPS-treated mice (Fig. 2D). Moreover, 4-OI also increased the levels of Nrf2, HO-1, and NQO1, and partially reversed the increased level of IL-1β protein and *Il6* mRNA in LPS-induced BUMPT cells (Fig. 2E F, and 2G). Meanwhile, mitochondrial ROS (mROS) production induced by LPS was partially diminished by 4-OI (Fig. 2H and I). All these data reveal that 4-OI relieves LPS-induced renal oxidative stress and inflammatory response *in vivo* and *in vitro*.

### 4-OI promoted the LPS-induced mitophagy in tubular cells *in vivo* and *in vitro*

**Fig. 3:**
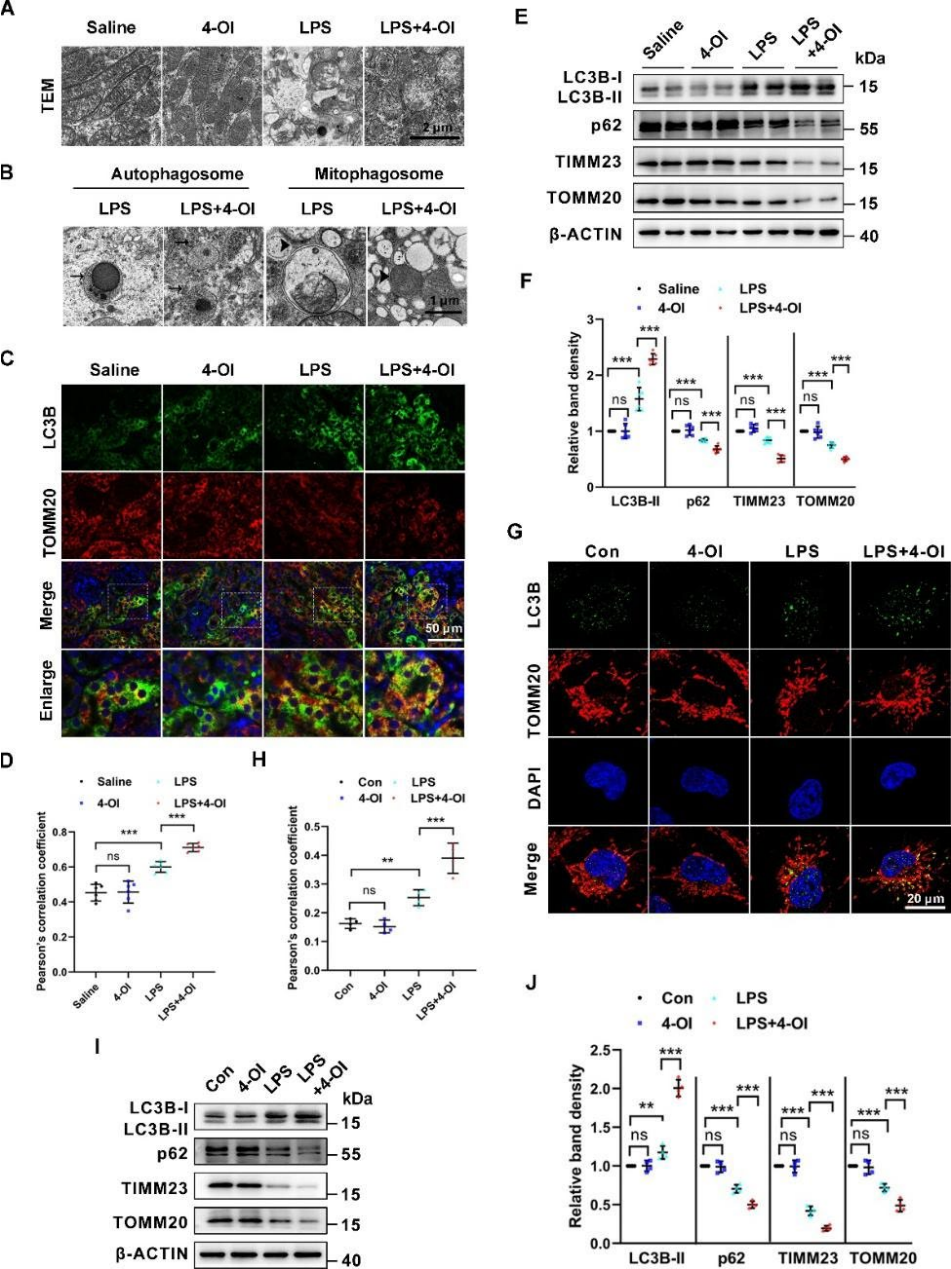
**4-OI ameliorates renal mitochondrial injury and improves mitophagy in LPS-induced AKI in mice and tubular cells. (A)** Mitochondria in proximal tubules were examined by TEM. Scale bar: 2 μm. **(B)** Representative TEM images of autophagosomes (pointed to by the arrowhead in the left two panels) and mitophagosomes (pointed to by the arrowhead in the right two panels) in renal proximal tubule cells. Scale bar: 1 μm. **(C)** Representative immunofluorescence images of LC3B (green) and TOMM20 (red) in kidney tissues from each group. The nuclei were counterstained by DAPI (blue) (n = 6). Scale bar: 50 μm. **(D)** Colocalization of LC3B and TOMM20 determined by Pearson’s correlation coefficient (n = 6). **(E)** Representative western blot bands of LC3B-II, p62, TIMM23, and TOMM20 in kidney cortices (n = 6). **(F)** Relative band intensity of LC3B-II, p62, TIMM23, and TOMM20 in kidneys (n = 6). **(G)** BUMPT cells were immunostained with LC3B (green) and TOMM20 (red) to show mitophagy (n = 4). Scale bar: 20 μm. **(H)** Colocalization of LC3B and TOMM20 determined by Pearson’s correlation coefficient (n = 4). **(I)** Representative western blot bands of LC3B-II, p62, TIMM23, and TOMM20 in BUNPT cells (n = 4). **(J)** Relative band intensity of LC3B-II, p62, TIMM23, and TOMM20 (n = 4). Each symbol (circle) represents an independent experiment. Data are presented as means ± SDs. *P < 0.05, **P < 0.01, ***P < 0.001; ns, not significant. TEM, transmission electron microscopy.

Mitophagy was reported to maintain mitochondrial homeostasis in S-AKI [33]. Therefore, we examined the effect of 4-OI in mitophagy in tubular cells *in vivo* and *in vitro*. We first detected the mitochondrial morphology and mitophagy in proximal tubular cells of the kidneys by transmission electron microscopy (TEM). As shown in Fig. 3A, the tubular mitochondria were elongated in control mice, while in LPS-induced mice were rounder and smaller. In addition, mitochondria in the LPS group were severely injured showing loss of cristae, fragmentation, swelling, and vacuoles in the mitochondria matrix. These changes in mitochondria induced by LPS were partially rescued by administration with 4-OI. TEM also revealed autophagosome and mitophagosome formation in tubular cells after LPS treatment, the number of which was increased in kidney tissues from 4-OI-treated AKI mice (Fig. 3B). Meanwhile, colocalization between punctate microtubule-associated protein 1 light chain 3 beta (MAP1LC3B/LC3B) and TOMM20 (translocase of outer mitochondrial membrane 20 homologs), which indicated activation of mitophagy [34], was upregulated in tubular cells from LPS group compared to control mice, and the colocalization was dramatically enhanced in 4-OI treated LPS group compared to the LPS group (Fig. 3C and D). Furthermore, western blot analysis showed that the protein level of LC3B-II was upregulated, and the levels of the soluble autophagy receptor sequestosome 1 (SQSTM1/p62), TIMM23 (translocase of inner mitochondrial membrane 23), TOMM20 were downregulated in the LPS group compared to the control group. 4-OI further elevated the level of LC3B-II and decreased the levels of p62, TOMM20, and TIMM23 in renal cortex tissues compared to the LPS group (Fig. 3E F). Next, we examined the occurrence of mitophagy in BUMPT cells exposed to LPS. An increase of mitophagy was noted in BUMPT cells treated with LPS compared to control as reflected by the accumulation of punctate LC3B -TOMM20 positive dots, the collocated puncta were increased by additional treatment with 4-OI (Fig. 3G H). Consistently, immunoblotting showed that LPS induced an increase in LC3B-II, and a decrease in p62, TIMM23, and TOMM20 (Fig. 3I J), suggesting that mitophagy was induced. 4-OI further enhanced mitophagy in the LPS-treated group (Fig. 3I J). Collectively, all these data reveal that 4-OI promotes LPS-induced mitophagy in tubular cells *in vivo* and *in vitro.*

### 4-OI attenuates LPS-stimulated renal injury by over-activating the Nrf2 pathway

**Fig. 4:**
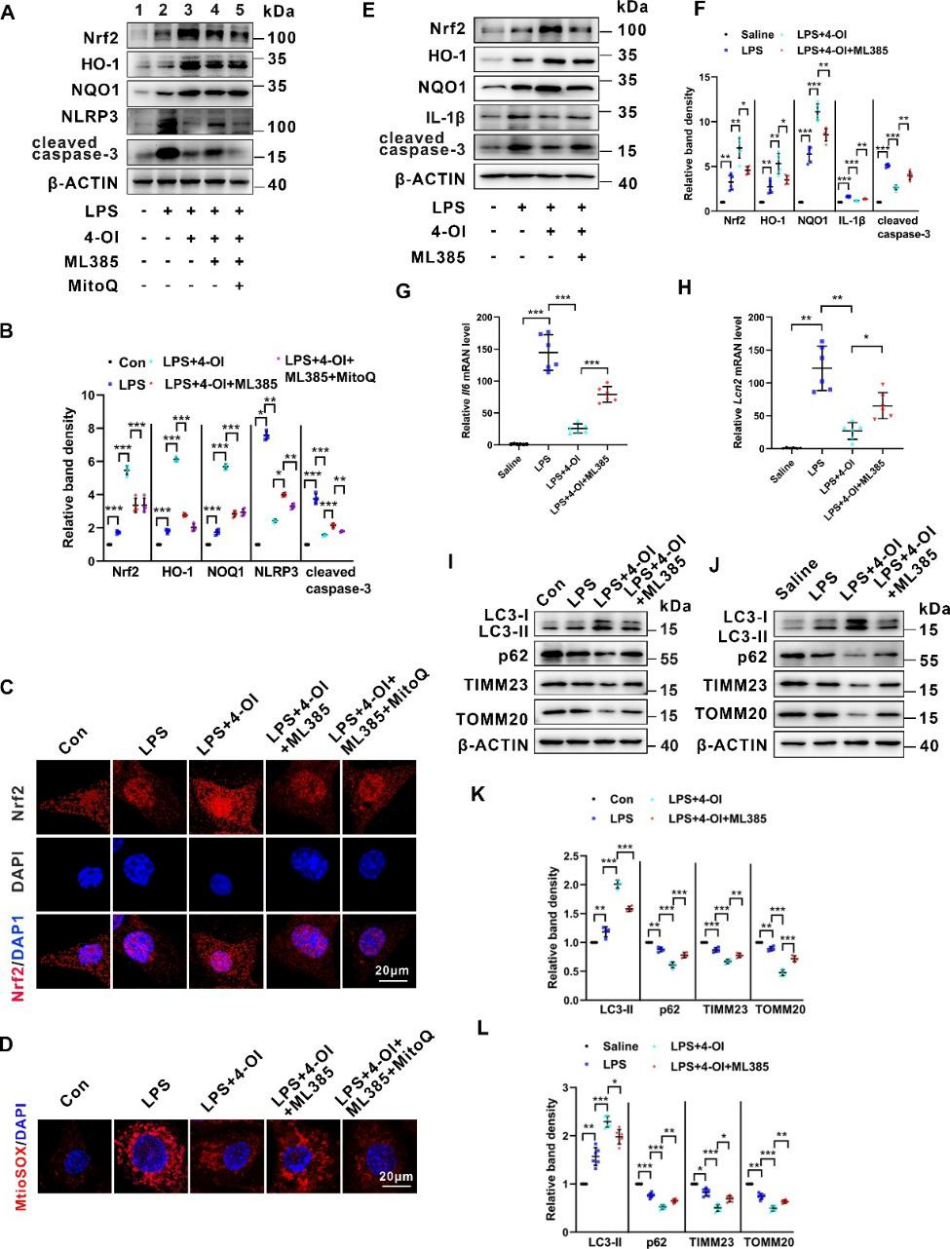
**4-OI attenuates LPS-induced mitochondrial ROS generation, and inflammation enhances mitophagy and subsequent apoptosis, which were partially abolished by the Nrf2 inhibitor. (A)** Representative western blot bands of Nrf2, HO-1, NQO1, NLRP3, and cleaved caspase-3 in BUMPT cells. **(B)** Relative band intensity of Nrf2, HO-1, NQO1, NLRP3 and cleaved caspase-3 in BUMPT cells (n = 4). **(C)** Representative IF staining images of Nrf2 (red) in BUMPT cells. Scale bar: 20 μm. **(D)** Representative images of MitoSOX staining of BUMPT cells. Scale bar: 20 μm. **(E)** Representative western blot bands of Nrf2, HO-1, NQO1, IL-1β, and cleaved caspase-3 in murine kidneys. **(F)** Relative band intensity HO-1, NQO1, IL-1β and cleaved caspase-3 in murine kidneys (n = 6). **(G)** qPCR analysis of mRNA of *Il6 * in renal tissues (n = 6). **(H)** qPCR analysis of mRNA of *Lcn2 * in renal tissues (n = 6). **(I)** Representative western blot bands of LC3B-II, p62, TIMM23, and TOMM20 in BUMPT cells (n = 4). **(J)** Representative western blot bands of LC3B-II, p62, TIMM23, and TOMM20 in renal tissues. **(K)** Relative band intensity of LC3B-II, p62, TIMM23, and TOMM20 in BUMPT cells (n = 4). **(L)** Relative band intensity of LC3B-II, p62, TIMM23, and TOMM20 in renal tissues (n = 6). Each symbol (circle) represents an independent experiment. Data are presented as means ± SDs. *P < 0.05, **P < 0.01, ***P < 0.001; ns, not significant

The Nrf2 signaling plays a crucial role in the anti-inflammatory and anti-oxidative systems. To identify whether the Nrf2 pathway involves in 4-OI-treated S-AKI, ML385, an Nrf2-specific inhibitor that interferes with the transcriptional activity of Nrf2, was used [35]. Additionally, to explore whether the effects of 4-OI are dependent on the anti-oxidative activity of Nrf2, we also used a mitochondria-targeted antioxidant, MitoQ. As western blot analysis shown in Fig. 4A and B, ML385 treatment partially blocked 4-OI-triggered the increase Nrf2, HO-1, and NQO1, and reversed the decreased levels of NLRP3 and cleaved caspase-3 in BUMPT cells challenged by LPS (Lane 4 vs. lane 3). Moreover, the changes induced by ML385 were partially dampened by pre-treated with MitoQ (Lane 5 vs. lane 4). Immunofluorescence staining further demonstrated Nrf2 was translocated into the nucleus induced by LPS, and 4-OI enhanced these changes in BUMPT cells. ML385 partially inhibited the nuclear localization of Nrf2 induced by 4-OI, while MitoQ could reverse this effect of ML385 (Fig. 4C). As MitoSox Red staining showed in Fig. 4D, mROS production induced by LPS was decreased by 4-OI treatment, ML385 partially restrained the changes induced by 4-OI, and the effects of ML385 were partially blocked by MitoQ. Furthermore, in LPS-treated mice, 4-OI upregulated the protein levels of Nrf2, HO-1, and NOQ1, and downregulated IL-1β in the kidneys. ML385 partially impaired these changes induced by 4-OI (Fig. 4E F). qPCR suggested that 4-OI repressed the *Il6* mRNA level induced by LPS in the renal tissues, and ML385 partially restrained the effect of 4-OI (Fig. 4G). Additionally, qPCR further confirmed that ML385 treatment increased the *Lcn2* level which was decreased by 4-OI (**4 H**). On the other hand, immunoblots showed that ML385 inhibited 4-OI-induced mitophagy as evidenced by restraining the protein levels of LC3B-II and enhancing p62, TIMM23, and TOMM20 in BUMPT cells (Fig. 4I K). Corresponding protein changes of mitophagy were observed in the kidneys of mice treated with ML385, as compared to BUMPT cells (Fig. 4J L). Taken together, these results indicate that 4-OI attenuates LPS-stimulated renal injury by over-activating the Nrf2 pathway.

### 4-OI inactivates the STAT3 pathway involved in LPS-induced AKI

**Fig. 5:**
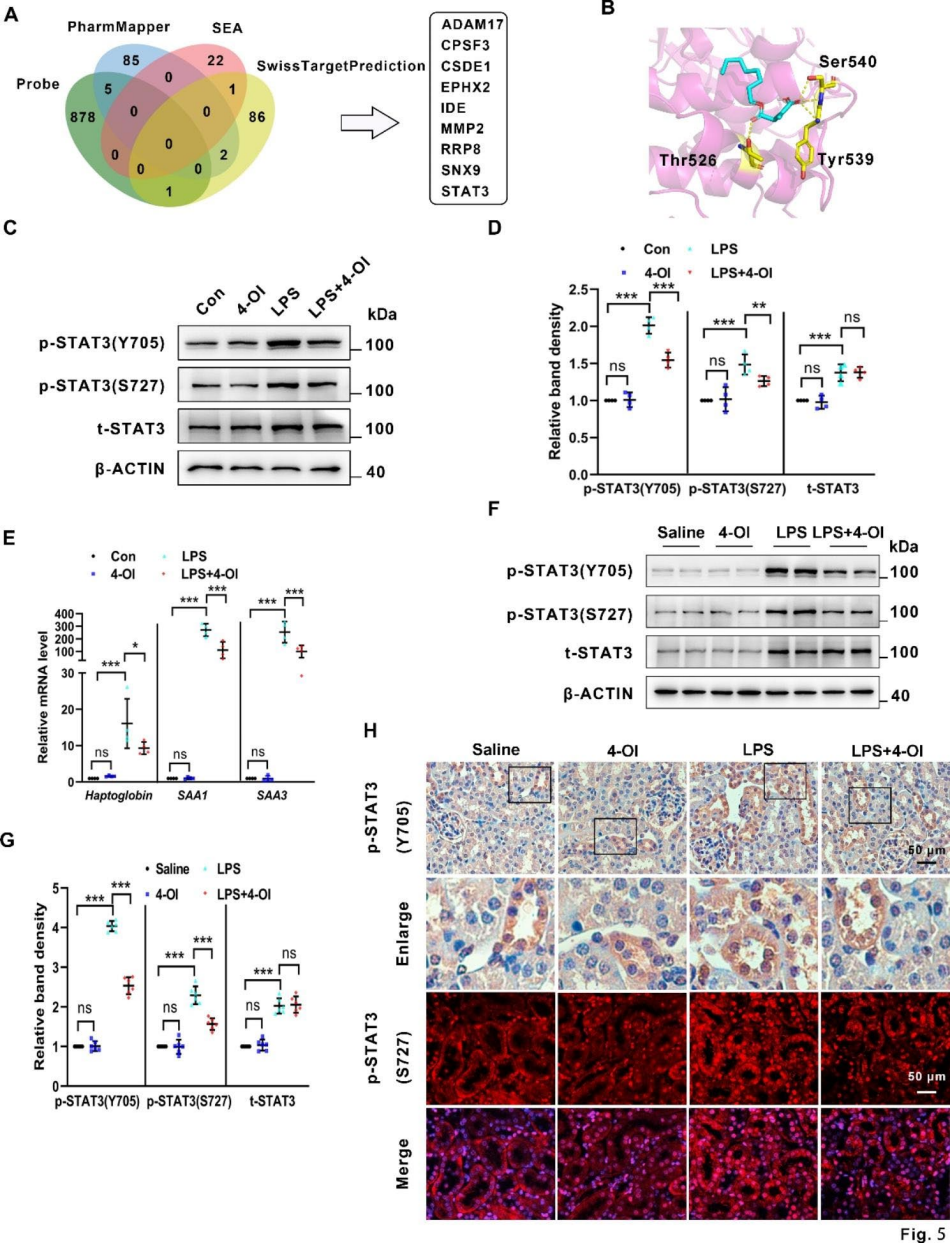
**Identified STAT3 as a potential target of 4-OI which was suppressed by 4-OI in LPS-induces AKI models. (A)** Venn diagram composed of the number of proteins modified by the specific bioorthogonal probe, PharmMapper database retrieved 4-OI targets, SEA database retrieved 4-OI targets and SwissTargetPrediction database retrieved 4-OI targets. **(B)** Molecular docking of 4-OI with STAT3. The pink structures in the panel represent screened macromolecular receptor structures, while the yellow sticks represent the interacted residuals, and the yellow dotted line represents intermolecular hydrogen bonding. **(C)** Representative western blot bands of p-STAT3 (Y705), p-STAT3 (S727), and total STAT3 in BUMPT cells. **(D)** Relative band intensity of p-STAT3 (Y705), p-STAT3 (S727), and total STAT3 in BUMPT cells (n = 4). **(E)** Real-time polymerase chain reaction analysis of mRNA of *Haptoglobin*, *SAA1*, and *SAA3* in BUMPT cells (n = 4). **(F)** Representative western blot bands of p-STAT3 (Y705), p-STAT3 (S727), and total STAT3 in renal tissues (n = 6). **(G)** Relative band intensity of p-STAT3 (Y705), p-STAT3 (S727), and total STAT3 in renal tissues (n = 6). **(H)** Representative IHC staining images of p-STAT3 (Y705) and IF staining images of p-STAT3 (S727) (red) in renal tissues. Scale bar: 50 μm. Each symbol (circle) represents an independent experiment. Data are presented as means ± SDs. *P < 0.05, **P < 0.01, ***P < 0.001; ns, not significant. SEA, Similarity ensemble approach

4-OI has been reported to exert multi-target activities [36, 37]. To explore the new targets of 4-OI, we used the PharmMapper databases, Similarity ensemble approach (SEA), and SwissTargetPrediction databases, and found plenty of potential targets of 4-OI. Besides, Wei Qin et al. developed a specific bioorthogonal probe, itaconate-alkyne, for quantitative and site-specific chemoproteomic profiling of itaconate in inflammatory macrophages [38], which delineated 884 potential itaconate substrates. As shown in Fig. 5A, after comparing the substrates from the above databases and research, nine substrates were found to be overlapped (ADAM17, CPSF3, CSDE1, EPHX2, IDE, MMP2, RRP8, SNX9, and STAT3). Among these candidates, STAT3 has been reported to be involved in the pathogenesis of sepsis [39] and S-AKI [40]. Upon LPS stimulation, STAT3 was phosphorylated at Tyr^705^ and Ser^727^ (p-STAT3(Y705) and p-STAT3(S727)) and then translocated to the nucleus to regulate the target genes’ expression. Therefore, we proposed that STAT3 may be a potential target of 4-OI in septic AKI. Next, Autodock vina (version 1.1.2) and PyMol (version 2.4.0) were utilized to obtain the best docking poses and binding affinities of 4-OI and STAT3. Autodock vina showed that the highest binding affinities between 4-OI and STAT3 were − 5.4 kcal/mol. The key amino acid residues of the 4-OI-binding site were Thr526, Tyr539, and Ser540 (Fig. 5B). Furthermore, the western blot showed that the levels of p-STAT3(Y705), p-STAT3(S727), and total STAT3, were upregulated in BUMPT cells induced by LPS. 4-OI partially reversed the changes of p-STAT3(Y705) and p-STAT3(S727), and did not change the level of total STAT3 (Fig. 5C and D). Additionally, qPCR demonstrated that LPS induced an increase of mRNAs of the target genes of STAT3, such as *haptoglobin*, *SAA1*, and *SAA3* (fold changes of 16, 270, and 250, respectively), all of which were partially retracted by 4-OI treatment (fold changes of 9.3, 112 and 100, respectively) (Fig. 5E). Next, as shown by the western blot of renal tissues, the changes of p-STAT3(Y705), p-STAT3(S727), and total STAT3 were compatible with that in BUMPT cells (Fig. 5F and G). Furthermore, IF and IHC staining of the kidney cortex demonstrated a notable accumulation of phosphorylated STAT3 in the nucleus of tubular cells in the LPS group indicating activation of STAT3, while these changes were reversed by 4-OI treatment (Fig. 5H).

### STAT3 overexpression diminishes 4-OI-induced effects in LPS-treated BUMPT cells

**Fig. 6:**
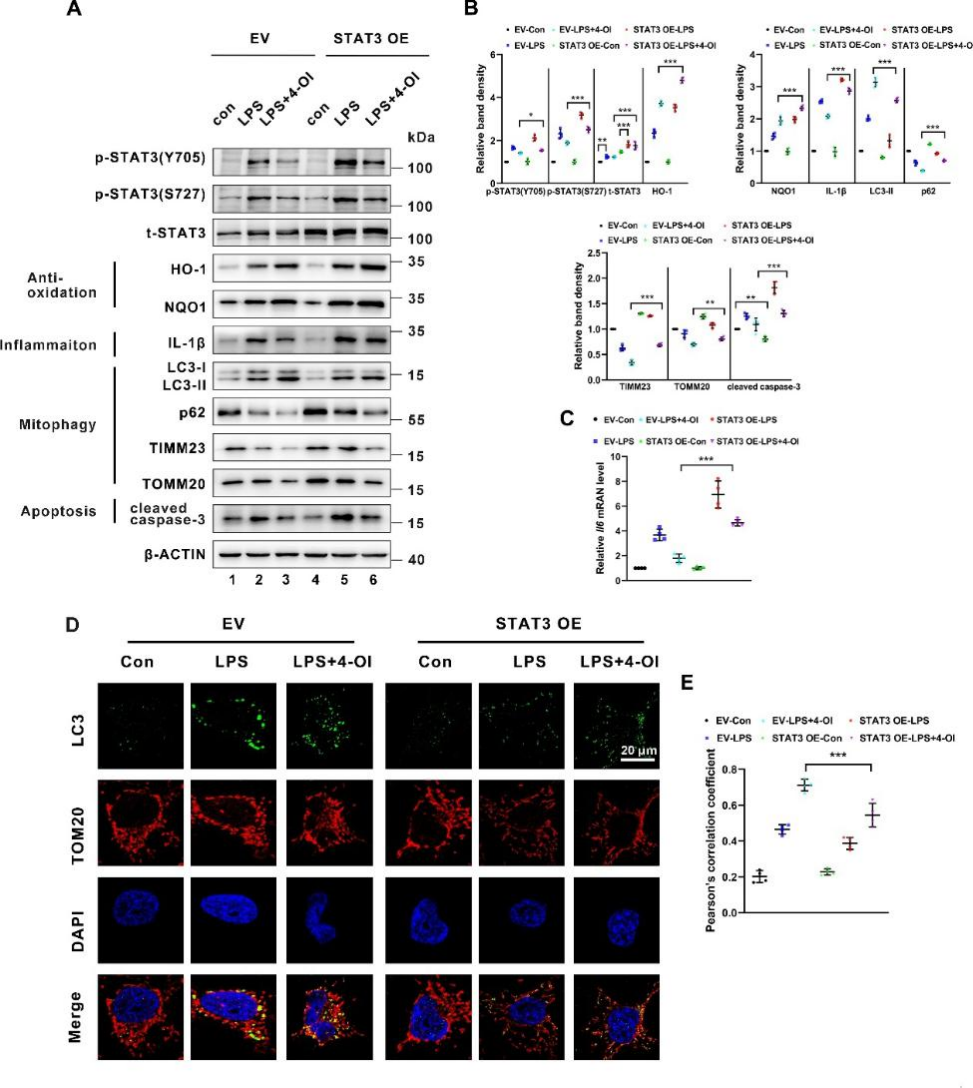
**Transfection with STAT3 plasmid attenuated 4-OI-induced anti-inflammatory effect and mitophagy in BUMPT cells treated with LPS. (A)** Representative western blot bands of p-STAT3 (Y705), p-STAT3 (S727), total STAT3, HO-1, NQO1, IL-1β, LC3B-II, p62, TOMM20, TIMM23, and cleaved caspase-3 in BUMPT cells (n = 4). **(B)** Relative band intensity of p-STAT3 (Y705), p-STAT3 (S727), total STAT3, NLRP3, HO-1, NQO1, p62, TOMM20, TIMM23, LC3-II and cleaved caspase-3 in BUMPT cells (n = 4). **(C) **qPCR analysis of mRNA of *Il6 * in BUMPT cells (n = 4). **(D)** BUMPT cells were immunostained with LC3B (green) and TOMM20 (red) to show mitophagy (n = 4). Scale bar: 20 μm. **(E)** Colocalization of LC3B and TOMM20 determined by Pearson’s correlation coefficient (n = 4). Each symbol (circle) represents an independent experiment. Data are presented as means ± SDs. *P < 0.05, **P < 0.01, ***P < 0.001; ns, not significant. EV, empty vector; STAT3 OE, STAT3 overexpression

To further confirm that 4-OI reverses the effect on LPS-induced BUMPT cells through STAT3, firstly, we transfected BUMPT cells with STAT3 plasmid (STAT3 OE), and stimulated with LPS and 4-OI or not. As shown in Fig. 6A and B, the protein levels of HO-1, NQO1, and IL-1β were further upregulated in the STAT3 OE group compared to the empty vector (EV) group when subjected to LPS and 4-OI (Lane 6 vs. lane 3). Furthermore, an increase of autophagy and mitophagy was noted in BUMPT cells treated with LPS and 4-OI compared to the LPS group as reflected by the increase of LC3B-II and reduction of p62, TOMM20, and TIMM23 protein levels, both of which were downregulated by overexpressing STAT3 (Fig. 6A, Lane 6 vs. lane 3 and B). This finding was further confirmed by IF staining, in which 4-OI enhances the punctuate LC3B associated with TOMM20 in the LPS group, and overexpression of STAT3 dramatically reversed the mitophagy (Fig. 6D and E). *Il6 * mRNA level was also upregulated in the STAT3 OE group compared to the EV group when subjected to LPS and 4-OI (Fig. 6C). Moreover, 4-OI alleviated LPS-induced apoptosis reflected by the reduction of cleaved caspase-3 measured by western blot. This effect was partially diminished by transfecting with STAT3 plasmid (Fig. 6A Lane 6 vs. lane 3 and B). To sum up, these observations indicate that 4-OI may enhance mitophagy and suppress inflammation through attenuating STAT3 activation, and the antioxidative effect of 4-OI might be independent of STAT3 activation in LPS-induced tubular cells.

## Discussion

In this study, we demonstrated for the first time that 4-OI improves renal function and alleviates injury of tubular cells in S-AKI via activating the Nrf2 pathway and blunting the STAT3 overactivation, indicating that 4-OI might be a promising therapeutic candidate for the treatment of sepsis-induced AKI in the future.

Mills, E. L., and collaborators first reported that 4-OI was a novel itaconate derivative [19]. The cell membrane–permeation of 4-OI makes it a suitable exogenous substitution to explore the effect of itaconate enrichment in the cells. Moreover, 4-OI is hydrolyzed to itaconate making it more appropriate to mimic endogenous itaconate than another itaconate derivative, such as dimethyl itaconate (DMI), since DMI is not metabolized into itaconate intracellularly [41]. More importantly, a series of studies manifested that 4-OI had a wide range of pharmacological effects, such as anti-inflammatory, antioxidative, and antifibrotic, and was used for the treatment of many animal models, such as acute lung injury [42], urate-induced peritonitis [37], cartilaginous endplate degeneration [43] and so on. Here, we revealed that 4-OI attenuated LPS-induced kidney dysfunction by protecting tubular cells from injury (Fig. 1).

The Nrf2 system is the central modulator of cellular and organismal to maintain oxidative homeostasis. Under quiescent conditions, KEAP1 represses Nrf2 by facilitating ubiquitinated Nrf2 through proteasome-mediated degradation, which maintains Nrf2 at low levels. When exposed to ROS, the KEAP1-CUL3 complex is disrupted, which causes Nrf2 to translocate to the nucleus to activate a battery of anti-oxidative genes, such as HO-1, and NQO1, defending against oxidative stress [44]. Here, we found that by the LPS challenge, the total and nuclear Nrf2 in both renal tissues and tubular cells was increased and the downstream targets such as HO-1 and NQO1 were upregulated (Fig. 2 and Fig. 4). These alterations of Nrf2 signaling may be a self-protective mechanism to maintain cellular homeostasis in response to LPS-induced ROS production. However, it was also reported that the total Nrf2 was unaltered but the nuclear Nrf2 was upregulated after LPS treatment [45]. The distinction between these results and ours may be due to the different animal models used. Furthermore, we showed that 4-OI drove further activation of the Nrf2 signaling and subsequent cytoprotective Nrf2-dependent expression of genes to restore the homeostatic balance in LPS-treated tubular cells, which was confirmed by using an Nrf2 inhibitor (Fig. 4). Additionally, western blot did not reveal any significant differences in the protein levels of KEAP1 among each group (data not shown). These results were in line with the previous research [19]. Together, these data indicate that 4-OI plays a protective role in tubular injury induced by LPS through activating the Nrf2 pathway.

4-OI plays complex and pleiotropic roles in different diseases. Ke Liu and colleagues reveal that 4-OI increases autophagic response in carboplatin-resistant Y79 cells [46]. 4-OI also promoted autophagy in osteoarthritis [47]. On the contrary, 4-OI ameliorated renal fibrosis by inhibiting autophagy in transforming growth factor-β1 (TGF-β1) induced-HK-2 cells [48]. In our study, we showed that 4-OI upregulated mitophagy and protected renal tubular cells following LPS stimulation (Fig. 5). The different roles of 4-OI in autophagy may depend on the cell types and context.

The role of dysregulated mitophagy in renal diseases, particularly S-AKI [49], is well established, and nonspecific chemical chaperones or cellular therapies, such as dexmedetomidine (DEX) [50] and bone marrow-derived mesenchymal stem cells (BMSCs) [18], have been shown to induce enhanced mitophagy, attenuating experimental S-AKI. However, the therapeutic approaches that conquered the S-AKI are far from well-established. In our current study, we showed that 4-OI enhanced mitophagy in LPS-induced AKI (Fig. 3). But the mechanism of how 4-OI regulates autophagy is poorly understood. One possible mechanism is due to the 4-OI-induced activation of Nrf2 [19]. Intranuclear of Nrf2 promotes the overexpression of autophagy-related genes resulting in enhanced autophagy [51–54]. Moreover, another precious mechanism underlying the regulation of autophagy is the alteration of STAT3. Our study indicated a novel aspect that overexpression of STAT3 impaired the 4-OI-induced mitophagy (Fig. 6). In the previous research, Tae Woo Kim et al. found that the reduction of STAT3 phosphorylation induced the activation of autophagy [55]. Eijiro Yamada et al. showed that STAT3Y705F mutant markedly increases autophagic flow in the WT tibialis anterior muscle [56]. Moreover, Xue Rong found that the down-regulation STAT3 phosphorylation is responsible for the induction of macrophage autophagy [57]. In sum, nuclear STAT3 may execute anti-autophagic functions and this may shed light on how 4-OI induced mitophagy by suppressing STAT3 phosphorylation.

We also explored potential target proteins of 4-OI in some databases and found that STAT3 may be a potential target of 4-OI. Moreover, Wei Qin and colleagues delineate that STAT3 may be a substrate of itaconate [38]. Besides, a series of studies indicated that STAT3 is activated in LPS-induced AKI, and downregulated STAT3 could attenuate tubular injury [58, 59]. Hence, we focused on the alteration of STAT3 in the tubular cells of the LPS-induced AKI model and found that 4-OI could suppress the upregulation of LPS-induced p-STAT3(Y705) and p-STAT3(S727) to protect renal tubular cells in the septic state (Fig. 5). Hence, we speculate that 4-OI may target STAT3 to inhibit the phosphorylation of STAT3. Moreover, mutants of the STAT3 linker domain (LD) have profound effects of inhibiting STAT3 transcriptional activation [60]. Additionally, astaxanthin, a xanthophyll carotenoid compound, binds to the LD to block STAT3 activity [61]. HO-1 [62], and CREBZF [63] binds to the LD to exert its inhibitory effect on STAT3. We predict that the key amino acid residues of the 4-OI-binding site were Thr526, Tyr539, and Ser540 (Fig. 5), which are located in the linker domain (LD) of STAT3. Our data suggest that 4-OI may bind to the LD of STAT3 to inhibit its activity. However, the molecular structure through which 4-OI inhibits STAT3 phosphorylation remains to be delineated in future studies.

## Conclusion

**Fig. 7:**
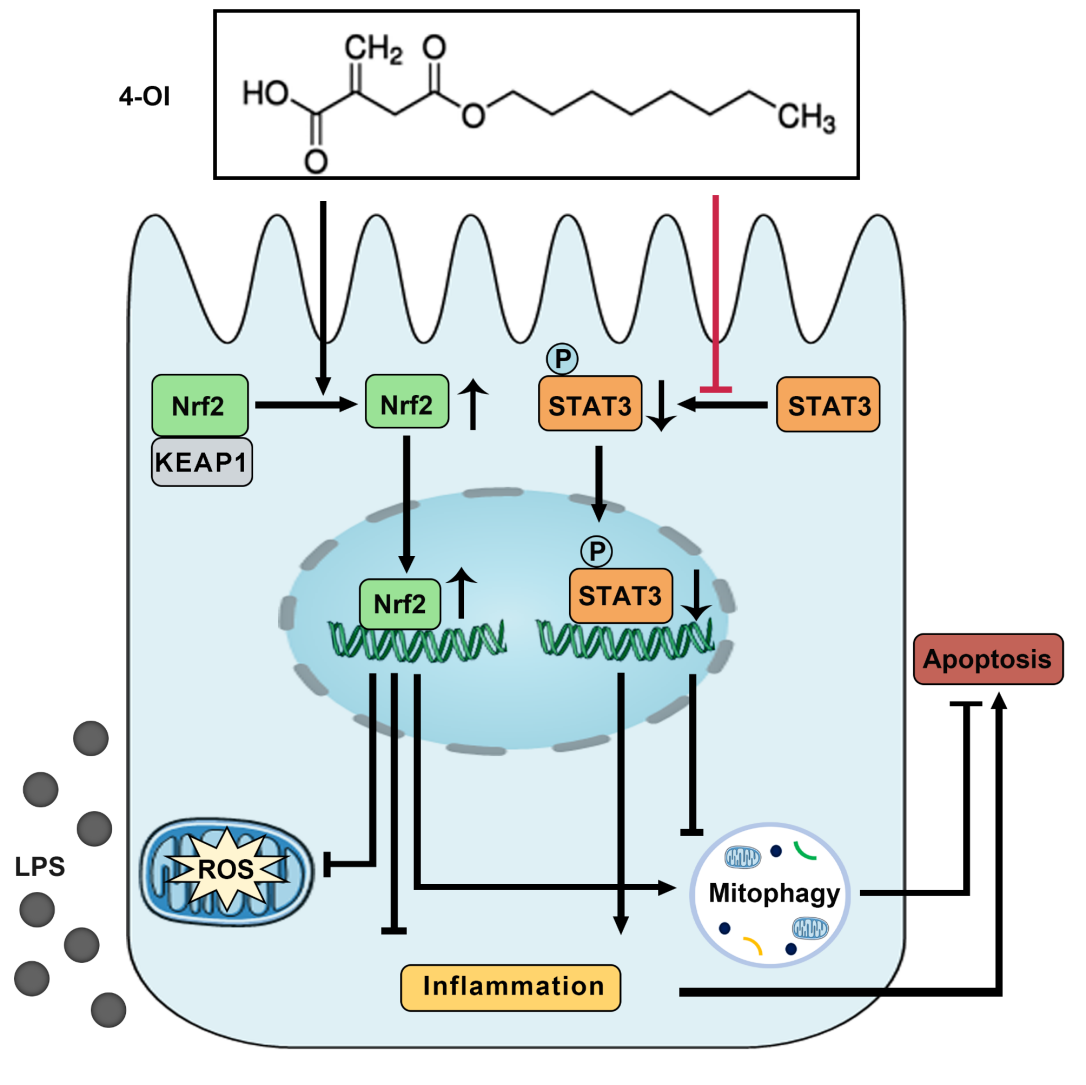
**A schematic diagram illustrating the protective effects of 4-OI against LPS-induced acute kidney injury.** 4-OI prevented LPS-induced acute kidney injury by over-activating Nrf2 and inactivating STAT3 signaling, attenuating oxidative stress, and inflammation, promoting mitophagy, and moderating apoptosis

In summary, this study demonstrates that 4-OI protects against septic stress in kidney tubular cells and tissues. 4-OI suppresses LPS-induced tubular cell inflammation and oxidative stress and enhances mitophagy *in vivo* and *in vitro*. Mechanistically, 4-OI up-regulates the expression of Nrf2 and activates Nrf2 signaling to execute anti-oxidative stress and anti-inflammatory response. Moreover, 4-OI may also target STAT3 to inactivate STAT3 signaling to affect mitophagy (Fig. 7). Thus, 4-OI may be a potential therapeutic approach for septic-AKI.

**Table 1:** PCR primer sequences

Genes	Direction	Sequence (5’-3’)
Haptoglobin	Forward	TATGGATGCCAAAGGCAGCTTTCC
	Reverse	TCGCTGTGGTTCAGGAAGAGGTTT
SAA1	Forward	GCGAGCCTACTCTGACATGAAG
	Reverse	CCCCCGAGCATGGAAGTATT
SAA3	Forward	CCTGGGCTGCTAAAGTCATCA
	Reverse	CCATGTCCCGTGAACTTCTGA
Lcn2	Forward	GCAGGTGGTACGTTGTGGG
	Reverse	CTCTTGTAGCTCATAGATGGTGC
Il6	Forward	TCCAGTTGCCTTCTTGGGAC
	Reverse	GTACTCCAGAAGACCAGAGG
ACTB	Forward	GGCTGTATTCCCCTCCATCG
	Reverse	CCAGTTGGTAACAATGCCATGT

## Electronic supplementary material

Below is the link to the electronic supplementary material.

Supplementary Material 1

Supplementary Material 2

Supplementary Material 3

## Data Availability

All data generated or analyzed during this study are included in this published article [and its supplementary information files].
