# Supplementary figures and images for: 4-Octyl itaconate attenuates LPS-induced acute kidney injury by activating Nrf2 and inhibiting STAT3 signaling
Source: Mol Med. 2023 Apr 24;29:58. doi: 10.1186/s10020-023-00631-8 (PMC10127401; doi:10.1186/s10020-023-00631-8)

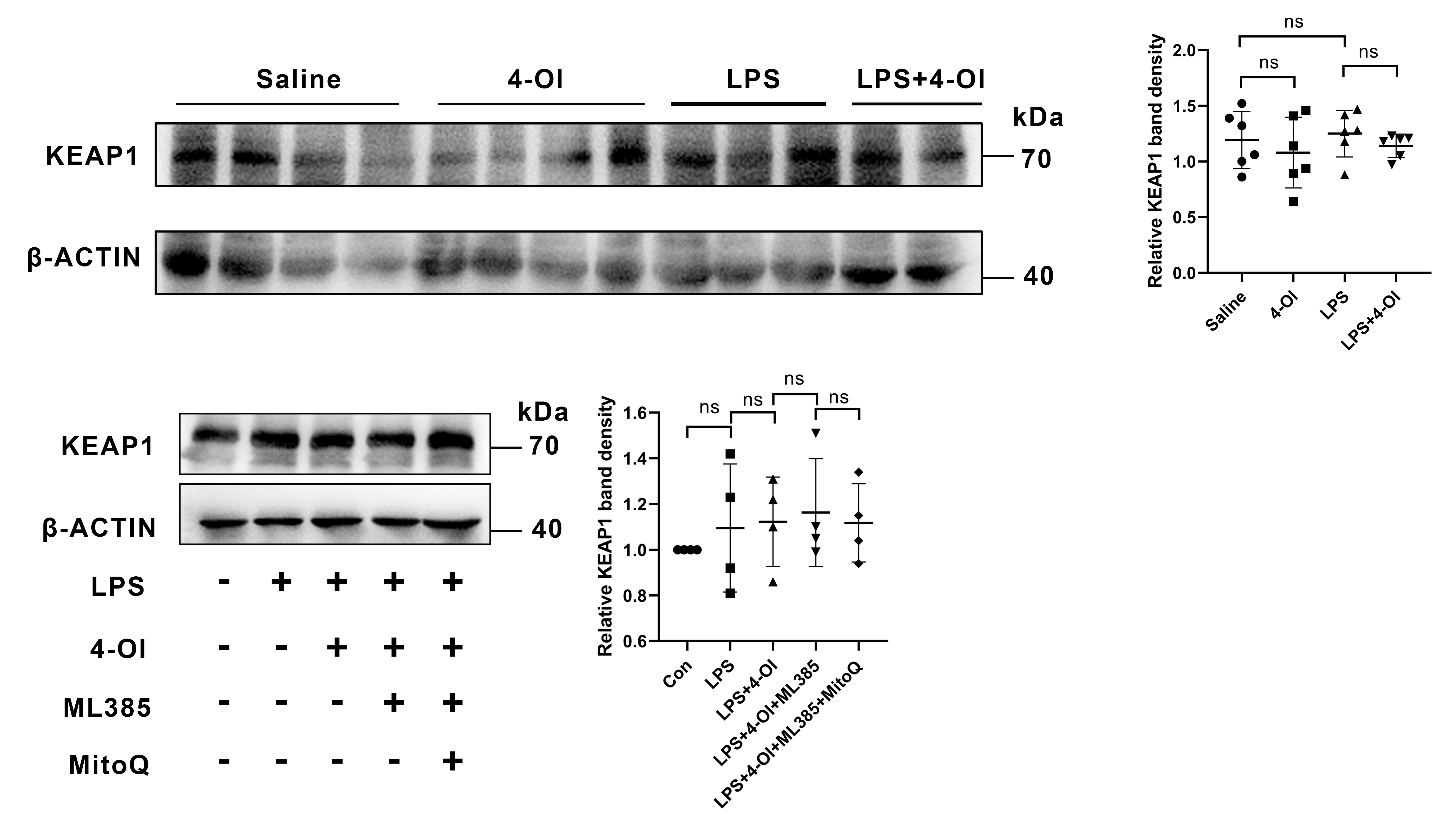

Supplement: Supplementary file 1 — Supplementary Material 1 [file 10020_2023_631_MOESM1_ESM.tif]
