# Supplementary material for: 4-Octyl itaconate attenuates LPS-induced acute kidney injury by activating Nrf2 and inhibiting STAT3 signaling
Source: Mol Med. 2023 Apr 24;29:58. doi: 10.1186/s10020-023-00631-8 (PMC10127401; doi:10.1186/s10020-023-00631-8)
Supplement: Supplementary file 3 — Supplementary Material 3 [file 10020_2023_631_MOESM3_ESM.docx]

**Response to the reviewers (MOME-D-22-01114)**

Firstly, we would like to thank the editors and reviewers for giving us constructive suggestions to improve the manuscript. We have carefully read the comments from the reviewers. Thank you very much for your valuable suggestions. According to the comments, we carried out additional experiments and checked the text. Please found the associated information below.

Reviewer reports:

Reviewer #1: General comments: In the present study, the authors examine the effects of 4-octyl itaconate (4-OI) on LPS-induced acute kidney injury. The 4-OI ameliorates LPS-induced AKI by suppressing inflammation, and oxidative stress, enhancing mitophagy through the activation of the Nrf2 signaling pathway, and inactivation of STAT3. Overall, the present study shows some interesting findings. However, there are some concerns needed to be addressed. My concerns are detailed below.

**Q1.** In figure 1C, what do the arrows in the image indicate? Please describe in the figure legend.

**A1.** Thank you very much. The arrows in the image indicate vacuolization and loss of brush border in injured tubular cells. We have described that in the **figure legend** of **Fig. 1C** in the revised manuscript. Please check it. Thank you.

**Q2.** In Figure 1E, it is nicely shown that BAX expression is suppressed by 4-OI treatment in kidney tissue, however, BAX expression was not examined in BUMPT cells (Figure 1I). Moreover, the authors should also examine BCL-2 expression both in vivo and in vitro.

**A2.** We agree with your comments and thanks for your question. Following your nice suggestions, we detected the associated protein expression using WB analysis. The results showed that 4-OI suppressed the increased BAX levels induced by LPS in BUMPT cells (**Fig. 1J of the revised manuscript**). In the kidneys of LPS-induced mice, BCL2 was decreased compared to the saline group, and 4-OI treatment partially reversed the changes of BCL2 (**Fig. 1F of the revised manuscript**). 4-OI also increased the BCL2 level in LPS-incubated BUMPT cells in a dose-dependent manner (**Fig. 1J of the revised manuscript**). Please check it in the revised manuscript. Thanks a lot.

**Q3.** In Figure 2, Nrf2 translocation data is not clear, you must be compared using a higher resolution of microscopy.

**A3.** Thanks a lot for your valuable suggestion. Following this question, we have updated the images with higher resolution. Please check it in **Fig 2A in the revised manuscript**. Thank you.

**Q4.** The authors examine the expression of nrf2. Why didn't the authors investigate keap1 expression? The authors should address this issue.

**A4.** This is a very professional suggestion and we thank you a lot. Under normal conditions, in the cytoplasm, KEAP1 forms a complex with CULLIN3 (CUL3) and polyubiquitinates NRF2, which marks NRF2 for rapid degradation through the proteasome system, and this constitutive degradation of NRF2 that maintains the quantity and activity of NRF2 at a low level. However, on exposure to electrophiles or ROS, the activity of the KEAP1-CUL3 complex was reduced and results in NRF2 stabilization. Nascent NRF2 can then directly translocate into the nucleus, bind to the ARE/EpRE, and robustly activate a battery of phase II genes, thereby acting as a master regulatory transcription factor (*Physiol Rev. 2018;98(3):1169-1203.*). 4-OI alkylates cysteine residues 151, 257, 288, 273, and 297 on the protein KEAP1, enabling Nrf2 to increase the expression of downstream genes with anti-oxidant and anti-inflammatory capacities (*Nature. 2018;556(7699):113-117.*). We detected the protein level of KEAP1 with WB. The results showed no significant differences among the saline, 4-OI, LPS, and LPS+4OI group. These results were in parallel with the previous research (*Nature. 2018;556(7699):113-117.*). Related sentences marked with red font and underlines have been added to the **Discussion** section of the revised manuscript. Thanks again.

**Q5.** In figure 3A and 3B, which part of the proximal tubule, S1, S2 or S3, is imaged in the TEM image? If possible, please provide low-magnification image.

**A5.** Thanks for your nice question. We did not mark the part of S1, S2 or S3 of the tubule in our images. The reasons are as follows. The S1, S2, and S3 designations were first defined based on ultrastructural characteristics in the kidneys of rats, and because of the variations of the ultrastructure among species, these terms are only properly applied to particular species. A morphometric study in mice found no structural segmentation in the cortical segments of the proximal tubule (*Arch Pathol. 1963; 76:290-302. Alan Yu, et al. Brenner and Rector's The Kidney 11th Edition. 2019; section 1, chapter 2, page 52.*). We also discussed this issue with the pathologist in our department, but unfortunately she was unable to differentiate between the S1, S2, or S3 segments based on the images we provided. Nevertheless, we will pay attention to this issue in future experiments. The low-magnification images are shown below. Thanks again.

**Q6.** All animals are survival during LPS-induced sepsis model?

**A6.** We would like to thank you for the comment. Yes, all animals survived in our LPS-induced sepsis model which consisted of some previous research showing that the mice can all survive in a dose of 10mg/kg (*Redox Biol. 2022;54:102367*), 15mg/kg (*JCI Insight. 2022;7(14):e158199. Acta Pharm Sin B. 2022;12(2):651-664.*) during 24h. Thank you again.

**Q7.** The authors measured the expression of LC3B-ǁ. Why did the authors quantify only 2 (Figure 3F, 3J, 4I, 4J, and 6B)?

**A7.** LC3B-I and LC3B-II are two forms of LC3. LC3-I is cytosolic, whereas LC3-II is membrane-bound (EMBO J. 2000;19(21):5720-8.). During autophagy, the soluble LC3-I converted to lipid bound LC3-II by ATG3 (*Nat Cell Biol. 2014;16(5):415-24.*), ATG7 (*Nature. 2009;461(7264):654-8. Autophagy. 2021;17(11):3402-3407.*) and the ATG12–ATG5–ATG16L system. The conversion of cytosolic LC3-I into membrane-bound LC3-II is indicative of autophagy induction and autophagosome formation (*EMBO J. 2000;19(21):5720-8. Nat Rev Nephrol. 2020;16(9):489-508.*). Therefore, we quantified only LC3B-II. Thanks a lot.

**Q8.** In figure 5H, why did the authors investigate the total-STAT3?

**A8.** We would like to thank the reviewer for this suggestion. The phosphorylated forms of STAT3 will be better to imply the activation of STAT3. Following your precious advice, we carried out a series of IHC and IF staining to detect the level of p-STAT3(Y705) and p-STAT3(S727) in renal tissues. We detected p-STAT3(Y705) and p-STAT3(S727) by IF first, however, p-STAT3(Y705) was not positive compared to the negative control. So, we reassessed p-STAT3(Y705) by IHC with the antibody diluted to 1:50. Results showed a notable accumulation of p-STAT3(Y705) and p-STAT3(S727) in the nucleus of tubular cells in the LPS group indicating activation of STAT3, while these changes were reversed by 4-OI treatment. Herein, we replaced the images in **Fig. 5H** **in the** **revised version**. Please check it. Many thanks again.

Reviewer #2: General comments: The present study showed 4-Octyl itaconate (4-OI) protected against LPS-induced acute kidney injury via activating Nrf2 and inhibiting STAT3 phosphorylation and activation to limit inflammation and oxidative stress, enhance mitophagy and improve renal tubular apoptosis. The authors utilized multiple in vivo and in vitro approaches to model LPS-induced kidney injury. The manuscript is well-written, the rationale is easy to follow, and conclusions are well supported by the data. There are some concerns listed below.

**Q1.** I am somewhat confused by the statistics paragraph in the methods. Did authors determine p value of multiple groups with ANOVA followed by post-hoc test or with ANOVA followed by a separate two-tailed T test? The former would be the proper statistical method so that there is an adjustment for multiple comparisons.

**A1.** We are thankful to you for your suggestions. We used ANOVA and post hoc tests to determine P values for multiple groups. When we use ANOVA to test the equality of at least three group means, statistically significant results indicate that not all of the group means are equal. However, ANOVA results do not identify which particular differences between pairs of means are significant. Thus, then we use post hoc tests to explore differences between multiple groups' means (*Multivariate Behav Res. 1980;15(3):281-97.*). Relevant sentences marked with red font and underlines have been replaced in the **Method** section of the **revised manuscript**. Thanks.

**Q2.** LPS at 10 mg/kg is typically a lethal dose of LPS. Did the authors lose mice during the 24 hour period? The authors are encouraged to perform a mortality curve of LPS and LPS + 4-OI and comment on mortality in their study as it would strengthen the manuscript*.*

**A2.** Thank you for your wonderful comment. We did not lose mice in our LPS-induced sepsis model. LPS at 10 mg/kg is lethal in some research. However, some studies also show that the mice can all survive in a dose of 10mg/kg (*Redox Biol. 2022;54:102367.*), 15mg/kg (*JCI Insight. 2022;7(14): e158199. Acta Pharm Sin B. 2022;12(2):651-664.*) during 24h. In our research, the survival rate of the LPS and LPS+4-OI group was 100% during 24h. Therefore, we did not perform the mortality curve. Thank you again.

**Q3.** Did the authors measure KIM1 or NGAL as additional injury markers in LPS murine models?

**A3.** Thank you for your nice suggestion. As suggested, we carried out q-PCR to measure the mRNA level of NGAL (*Lcn2*) in murine renal tissues (*Crit Care. 2021;25(1):202.*). Results showed that 4-OI reversed the increased level of *Lcn2* induced by LPS (**Fig. 1C of the revised version**) and ML385 impaired the changes induced by 4-OI (**Fig. 4H of the revised version**). The results were added in the revised version. Thanks a lot.

**Q4.** The resolution of images in Fig 1G is poor. Also, the authors should describe apoptotic index in the methods as they do in the Figure 1 legend.

**A4.** Thank you very much. As suggested, we have replaced the images in **Fig. 1H** **of the revised version** and added the apoptotic index in the **Methods** section marked with red font and underlines in the revised manuscript. Thanks again.

**Q5.** In Fig 2A, again the resolution is very poor so I cannot visualize the nuclear translocation of Nrf2 that the authors claim is occurring.

**A5.** Thanks a lot for your valuable suggestion. Following this question, we have updated the images with higher resolution. Please check it in **Fig 2A** **in the revised version**. Thank you.

**Q6.** Did the authors test any other inflammatory genes in addition to IL1b in Figs 4 and 6? STAT3 is known to regulate the IL6 pathway. Did they measure IL6? If not, then I would consider removing the IL1b data as it does not add much to findings.

**A6.** We thank the reviewer to point out that IL6 is more suitable and we agree with it. As suggested, we verified the mRNA level of *Il6* by q-PCR and added the results in **Fig. 2G, Fig. 4G, and Fig. 6C in the revised manuscript**. Please check it. Additionally, in some research, Il1b is used to reflect the level of pro-inflammatory cytokines in septic-AKI (*Br J Anaesth. 2021;127(4):577-586. Biomed Pharmacother. 2023;159:114286. Life Sci. 2020;262:118505. Kidney Int. 1995;48(5):1563-70.*). IL-1β could initiate IL-6/STAT3 signaling (*Int Immunol. 2011;23(11):701-12.*) and STAT3 phosphorylation is critical for interleukin 1 beta production in response to LPS *(Mol Immunol. 2009;46(8-9):1867-77.*). So, we kept the IL1B in the revised manuscript. Thanks a lot. If you insist that there is no need to show the IL-1B, please let us know and we will remove it. Thanks a lot.

**Q7.** Page 5, line 2: please change "symptom" to "condition

**A7**. Thanks for the reviewer’s kind suggestion. We have carefully checked through our paper and updated the word with red font and underline. Please check it on **Page 5** of the **Introduction** section **in the revised version**. Thank you again.

**Q8.** Page 5, line 19: please change "portal" to "partial"

**A8**. We appreciate the reviewer’s comment. We have replaced the word with red font and underlines. Please check it on **Page 5** of the **Introduction** section i**n the revised version**. Thank you very much.
